# Association between patient-reported HIV status and provider recommendation for screening in an opportunistic cervical Cancer screening setting in Jos, Nigeria

**DOI:** 10.1186/s12913-018-3700-y

**Published:** 2018-11-22

**Authors:** Jonah Musa, Chad J. Achenbach, Charlesnika T. Evans, Neil Jordan, Patrick H. Daru, Lifang Hou, Robert L. Murphy, Isaac F. Adewole, Melissa A. Simon

**Affiliations:** 1 Health Sciences Integrated PhD Program, Center for Healthcare Studies, Institute of Public Health and Medicine, Feinberg School of Medicine, Northwestern University, Chicago, USA; 2 Center for Global Health, Institute of Public Health and Medicine, Feinberg School of Medicine, Northwestern University, Chicago, USA; 3 Department of Obstetrics and Gynecology, Faculty of Medical Sciences, University of Jos, Plateau State, Nigeria; 4 Division of Infectious Diseases, Department of Medicine, Feinberg School of Medicine, Northwestern University, Chicago, USA; 5 Department of Preventive Medicine, Center for Health Care Studies, Global Health, Institute for Public Health and Medicine, Feinberg School of Medicine, Northwestern University, Chicago, IL USA; 6 Center of Innovation for Complex Chronic Healthcare (CINCCH), Department of Veterans Affairs, Edward Hines Jr. VA Hospital, Hines, IL USA; 7 Department of Psychiatry & Behavioral Science, Feinberg School of Medicine, Northwestern University, Chicago, USA; 8 Center for Population Epigenetics, Robert H. Lurie Comprehensive Cancer Center and Department of Preventive Medicine, Northwestern University Feinberg School of Medicine, Chicago, IL 60611 USA; 9 Federal Ministry of Health, Federal Secretariat Complex, Central Business District, Federal Capital, Abuja, Nigeria; 10 Department of Obstetrics and Gynecology, Preventive Medicine and Medical Social Sciences, Feinberg School of Medicine, Northwestern University, Chicago, USA

**Keywords:** HIV status, Recommendation, Provider-referral, Cervical Cancer screening, Opportunistic screening, Utilization, Nigeria

## Abstract

**Background:**

Cervical cancer screening (CCS) is an important health service intervention for prevention of morbidity and mortality from invasive cervical cancer. The role of provider recommendation and referral is critical in utilization of this services particularly in settings where screening is largely opportunistic. We sought to understand how patient-reported human immunodeficiency virus (HIV) infection status is associated with provider referral in an opportunistic screening setting.

**Methods:**

We performed a cross-sectional analysis of data on a sample of women who had received a CCS at the “Operation Stop” cervical cancer (OSCC) screening service in Jos, Nigeria over a 10-year time period (2006–2016). We used the de-identified records of women who had their first CCS to analyze the association between patient-reported HIV and likelihood of provider-referral at first CCS. We performed descriptive statistics with relevant test of association using Student t-test (t-test) for continuous variables and Pearson chi square or Fisher exact test where applicable for categorical variables. We also used a bivariable and multivariable logistic regression models to estimate the independent association of patient-reported HIV on provider referral. All statistical tests were performed using STATA version 14.1, College Station, Texas, USA. Level of statistical significance was set at 0.05.

**Results:**

During the 10-year period, 14,088 women had their first CCS. The reported HIV prevalence in the population was 5.0%; 95% CI: 4.6, 5.4 (703/14,088). The median age of women who were screened was 37 years (IQR; 30–45). Women who were HIV infected received more referrals from providers compared to women who were HIV uninfected (68.7% versus 49.2%), *p*-value < 0.001. Similarly, we found an independent effect of patient-reported HIV infection on the likelihood for provider-referral in the screened sample (aOR = 2.35; 95% CI: 1.95, 2.82).

**Conclusion:**

Our analysis supports the design of health systems that facilitates providers’ engagement and provision of necessary counseling for CCS in the course of routine clinical care. The practice of offering recommendation and referrals for CCS to women at high risk of cervical cancer, such as HIV infected women should be supported.

**Electronic supplementary material:**

The online version of this article (10.1186/s12913-018-3700-y) contains supplementary material, which is available to authorized users.

## Background

Of the half million new cases of invasive cervical cancer (ICC) reported globally each year, over 80% occur in Low-and Middle Income Countries (LMICs) [1]. Nigeria is one of these countries with a high burden of ICC incidence and mortality [2]. The Global Burden of Cancer 2013 ranked cervical cancer the 2nd most common in incidence and mortality among all cancers in Nigeria [3].

CCS is an important health care service intervention for reducing ICC incidence and mortality and its benefits are evident from data in developed countries, where organized CCS programs have resulted to a substantial decreases in ICC incidence and mortality [4–10]. However, in Nigeria and other LMICs where organized CCS programs are lacking, the opportunity to have a screening test likely depends on several factors ranging from availability of screening, offering screening recommendations by providers, to health system support to overcome barriers to accessing services. The literature on cancer screening suggest that it is a process of care, consisting of several steps and interfaces between patients, providers, and health care organizations [11]. In this context, screening rates are largely driven by strategies that limit the number of interfaces across organizational boundaries; recruiting patients, promoting referrals, and facilitate appointment scheduling; and promote continuous patient care [11]. The organizational capability of the health care system to address these boundaries likely explains higher CCS rates (83%) in the US [12], in comparison to Nigeria and other LMICs in sub-Saharan Africa with much lower CCS rates between 6 and 8% [13, 14].

Indeed, we have an established body of literature on the effectiveness of provider recommendation for screening on CCS participation [11, 15–18]. Also, we have evidence in Human Immunodeficiency Virus (HIV) infected populations, suggesting that women’s awareness that HIV infection increases the risk of ICC and having a strong provider-patient relationship were significant facilitators for CCS utilization [19]. Therefore, provider-patient discussions about CCS and offering referrals for such screening are critical because there is a high burden of HIV in Nigeria and also a high burden of HIV-associated precancerous abnormalities of the cervix and ICC in HIV infected population [20–22]. We, however, do not understand the relationship between patient-reported HIV infection and provider behavior in providing a CCS recommendation and referral during the care process particularly in settings where CCS is largely opportunistic. In brief, opportunistic screening is dependent on a woman or her healthcare provider taking the initiative to do a pap test [23]. Indeed, strategies to improve early detection of cervical cancer through screening have focused either on opportunistic screening requested by a provider or an individual, or organized CCS in which a defined population is contacted and invited to screen at regular scheduled intervals [24]. We therefore hypothesized that in opportunistic screening settings, women with reported HIV infection are more likely to receive a provider-referral for CCS than women who are HIV uninfected. This manuscript reports on patient-reported HIV status and other socio-demographic factors associated with provider-referral in women at first CCS in an opportunistic screening in Nigeria.

### Conceptual framework

**Fig. 1:**
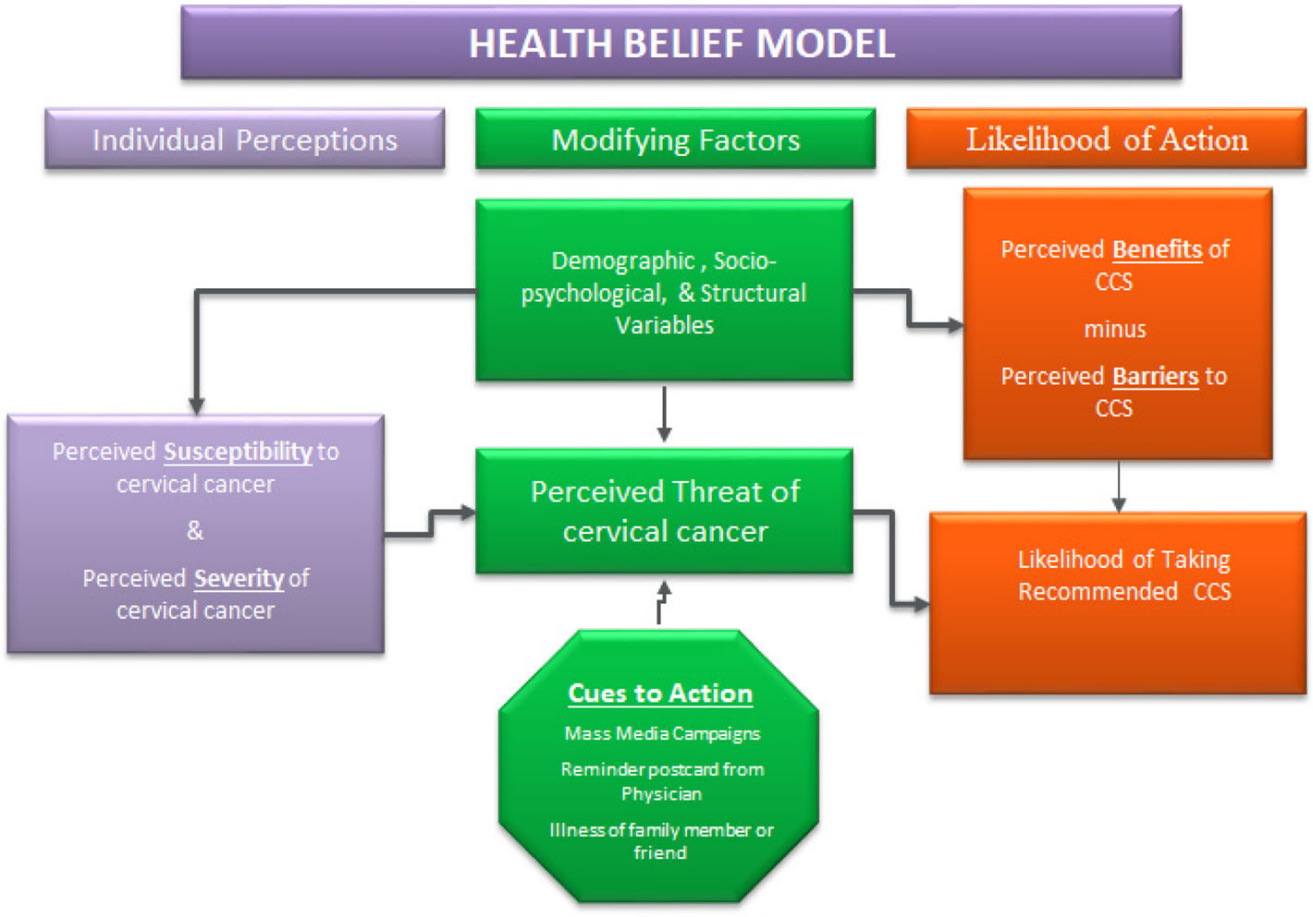
The Health Belief Model [25] (Adapted from Rosenstock, 1974)

**Fig. 2:**
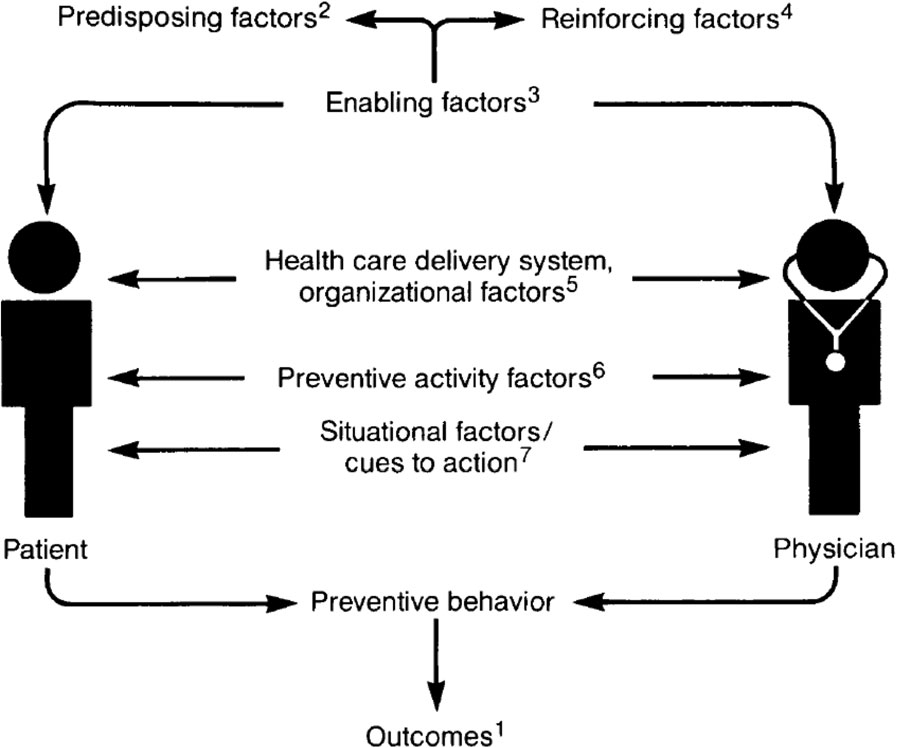
A Systems Model of Clinical Preventive Care: An Analysis of Factors Influencing Patient and Physician. In: Judith M.E. Walsh, *Health Educ Behav,* 1992 [26]. (1. Outcomes are defined as decreased disease incidence, decreased morbidity, and decreased mortality. 2. Predisposing factors related to the motivation to perform a particular health behavior. Patient predisposing factors include demographics; beliefs (health beliefs); attitudes; expectations; motivation (internal locus of control); self-efficacy; health value orientation. Physician predisposing factors include demographics; gender; ethnicity; language concordance; beliefs; attitudes; prior clinical experiences; and personal health preferences. 3. Enabling factors include education; health knowledge; skills; income; logistical factors; and physiologic factors. Physician enabling factors include training; technical expertise; knowledge; logistical factors; and availability of materials. 4. Reinforcing factors are those that support or reward the behavior. Patient reinforcing factors include social support/approval and inherent reinforcement value of the preventive activity. Physician reinforcing factors include patient satisfaction; support/approval of peers; and case finding. 5. Health care delivery system/organizational factors include access to care; availability of technology and personnel; organizational priorities; structure of the office practice; reimbursement; and coordination with community resources. 6. Preventive activity factors are features of the preventive activity itself and include costs; risks; efficacy; and effectiveness. 7. Situational factors/cues to action are triggers to health behavior and include internal cues, such as symptoms and external cues such as physician reminders

The “operation stop” cervical cancer (OSCC) screening unit at Jos, Nigeria has no formal system of inviting or recalling eligible women for CCS. Therefore, women either initiate the process of having a screening by coming to the unit (“self-referral”) or by recommendation for screening by a health care provider to eligible women (“provider-referral”. To understand the relationship between patient-reported HIV and provider recommendation and referral for CCS, we adapted the constructs of the Health Belief Model (HBM) [25] in Fig. 1 and the systems model of clinical preventive care [26] in Fig. 2. These models offer explanations on the provider’s role (“Cues-to-action”) in utilization of cervical cancer preventive services. These two models also explain the behavior of individual patients in taking and completing a screening behavioral action (perception of susceptibility, perception of seriousness of condition, perception of benefits of screening and ability to overcome barriers in the screening pathway- “self-efficacy”). The systems model of clinical preventive care recognizes the critical influence of physician-patient interaction and how situational and environmental factors in the health care system (cues-to-action) promotes preventive behavior towards cancer care [26].

The HBM was first described in the 1950s by a psychologist working in the US Public Health Service and has become one of the most widely used conceptual frameworks of health behavior [25]. The framework is based on the theory that people are afraid of getting serious illnesses, and that health-related behaviors are influenced by an individual’s level of fear, based on severity of threat perceived and the expected benefit of taking appropriate health behavioral action to avoid having the disease [25, 27]. For instance, “perceived susceptibility” helps our understanding of how patient-reported HIV status and other risk factors could influence patients’ decisions to seek CCS at an earlier age or prompt a provider to initiate risk counseling and offer a referral to have a CCS. Also, “Cue-to-Action” helps explain how provider-patient interaction could lead to a screening referral based on identified risk factors for cervical cancer during clinical care visits.

## Methods

### Study design and setting

We performed a cross-sectional analysis of data on a sample of women who had received a CCS at the “Operation Stop” cervical cancer (OSCC) screening unit in Jos, Nigeria over a 10-year time period (2006–2016). The OSCC unit commenced CCS and treatment in 2006 with funding from Exxon Mobil, Texas, USA, through the African Organization for Research and Training in Cancer (AORTIC). This project offered opportunistic CCS services to eligible women in Jos, neighboring towns, and states in northern Nigeria. Also, the project has maintained an up-to-date electronic database and backup paper records of women utilizing the service. This database has records of patient demographic and risk factor variables that are obtained from eligible women at the first screening visit prior to cervical sample collection for Pap smear test. Each participant is given a unique medical record number, and all subsequent records including the cytopathology reports are entered into a FileMaker Pro version 8.0 database [28].

### Study sample

**Fig. 3:**
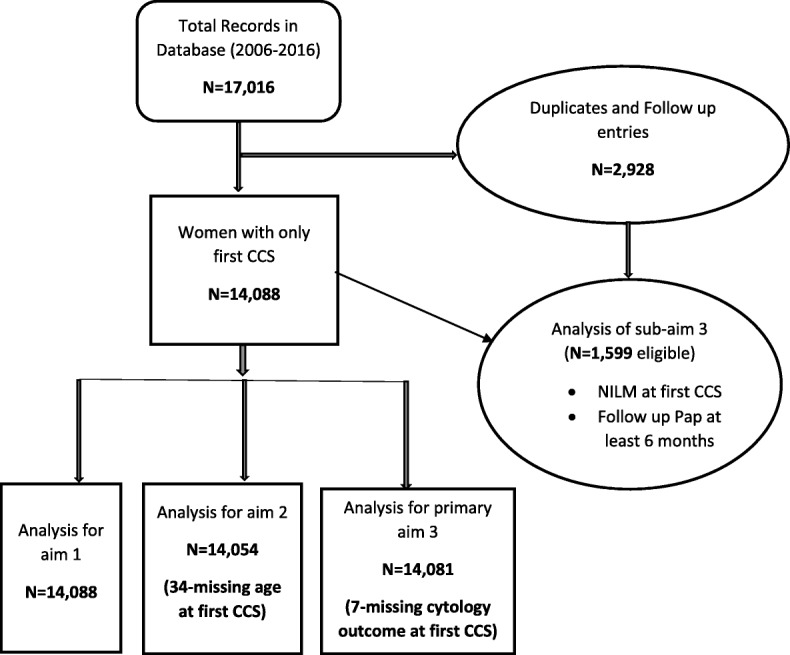
Study sample derivation for study aims 1, 2 and 3. Note: the subset for sub-aim 3 was derived from the primary sample of women with normal cervical cytology outcome at first CCS (NILM) and had at least one follow up cytology outcome (*N* = 1599). This manuscript represents the results of analysis for aim 1 as shown in the sample derivation flow

This study utilized de-identified patient data in the OSCC electronic database. We accessed the sociodemographic, risk factors and CCS cytology outcome variables for this analysis. Our source database included all women who had received CCS with cytology report documented in the database. We excluded follow-up entries and utilized only the records at the first CCS. The detailed description of the study sample derivation is illustrated in Fig. 3.

### Key independent and primary outcome variables

The electronic database has important variables ranging from age at first screening, source of referrals, patient-reported HIV status, sexually transmitted infections (STIs), age at first sexual debut, smoking history, alcohol consumption, reported lifetime number of sexual partners, parity, years of completed education, use of contraceptives and other risk factors. The primary outcome variable in this analysis was provider-referral for CCS. This variable is captured as binary: “yes” for provider-referral or “no” for self-referral. The principal independent variable was patient-reported HIV status. The operational definition of the variables has been described in Additional file 1.

### Statistical analysis

#### Descriptive statistics

We performed summary statistics on continuous and categorical variables of the study sample and obtained means, medians and proportions for the independent variables and outcome. We also compared the baseline characteristics of the sample with the primary outcome. Since the principal exposure variable in this analysis was patient-reported HIV status, we estimated the proportion of women who received a provider referral for CCS by patient-reported HIV status. We then performed a Pearson’s chi square test of the association between reported HIV status and provider-referral for CCS.

#### Bivariable and multivariable logistic regression model

To understand the independent effect of patient-reported HIV status on the likelihood of receiving a provider referral for a CCS, we evaluated the unadjusted association between HIV status and provider referral using logistic regression to get an unadjusted odds ratio (OR) and 95% confidence intervals (CI). A multivariable logistic regression analysis was performed to assess the independent effect of HIV status on provider referral for CCS adjusting for other characteristics. The adjusted odds ratio (aOR) and 95% CI were computed from the final model.

In the first step, we created a new binary variable “HIV status” from patient reported HIV to either “HIV infected” as “1” and “HIV not infected” as “0”. Women who did not know their HIV status were treated as “missing”. Similarly, we created indicator (dummy) variables from age at first screening, parity, number of lifetime sexual partners, education years completed, age at first sexual intercourse, reported history of vaginal infection and ever diagnosed with an STI. We also created a binary variable “referral group” with “provider-referral” as “1” and “self-referral” as “0”. The significant predictor variables associated with receiving a provider referral in the bivariable logistic regression analyses were included in a multivariable logistic regression model to estimate the independent effect of patient reported HIV infection on the likelihood of receiving provider referral in the study sample. Our final predictive model was selected using the backward selection method, and the model fit was assessed by the Hosmer-Lemeshow goodness-of-fit test. A *p*-value greater than 0.05 was considered a good model-fit [29]. We also considered the magnitude of change in the likelihood ratio chi square for each model before selecting the final model that best fits our data.

The reporting of the results presented in this manuscript was guided by the “strengthening the reporting of observational studies in epidemiology” (STROBE) guidelines for cross-sectional studies as indicated in the STROBE checklist (Additional file 2).

## Results

During the 10-year study period (2006 to 2016), there were 17,016 records of women who received CCS services using the Pap test at the OSCC. Since this cross-sectional analysis focused on data records of women at first CCS, multiple follow-up entries were excluded from this analysis (see Fig. 3 for details of sample derivation).

Therefore, a final study sample of 14,088 women was utilized for this analysis (Table 1 provides detailed descriptive characteristics of the sample population at first CCS). The median age at first CCS in the sample population was 37 years (IQR, 30–45) and a mean of 38.1 years ±10.1. A total of 703 out of 14,088 women reported their HIV status as infected leading to patient-reported HIV prevalence of 5.0% (95% CI: 4.6, 5.4). The proportion of women who received provider referral was 50.1% (95% CI: 49.2, 50.5), while women who received CCS by self-referral was 49.9% (95% CI: 49.1, 50.8). When we compared the proportion of women who received a provider referral by patient reported HIV status, we found that 68.7% (95% CI: 65.3, 72.1) of women with HIV received a provider referral compared to 49.2% (95% CI: 48.4, 50.1) of women who were HIV uninfected (*p*-value < 0.001). The baseline characteristics of the study sample is summarized in Table 2.

**Table 1:** Summary statistics of the socio-demographic and baseline cytology outcomes of women who received first CCS in an opportunistic cervical cancer screening program in Jos Nigeria (*N* = 14,088)

Characteristics	Descriptive statistics (Mean ± SD, Median, IQR or % in parentheses)	95% Confidence intervals
Age at first CCS	37; IQR, 30–45	
Age groups at first CCS		
< 21 years	1.1	1.0, 1.3
21–30	24.7	24.0, 25.4
31–40	37.3	36.5, 38.1
41–50	25.4	24.6, 26.1
51–60	8.9	8.5, 9.4
61–70	2.1	1.8, 2.3
≥ 71	0.2	0.2, 0.3
Missing	0.2	0.2, 0.3
Age at first sex	20; IQR, 18–22	
Education years completed	13; IQR, 12–14	
Annual household income in USD	3300; IQR, 1920-4800	
HIV status		
Infected	703 (5.0)	4.6–5.5
Not infected	13,155 (93.4)	93.0–93.8
Unknown (missing)	230 (1.6)	1.4–1.9
History of Vaginal infection		
Yes	80.0	79.4–80.7
No	16.6	16.0–17.2
Missing	3.4	3.1–3.7
Use of condoms		
Yes	7.4	6.8–7.6
No	86.2	85.6–86.8
Missing	6.6	6.2–7.1
Ever diagnosed with an STI		
Yes	10.0	9.5–10.5
No	60.8	60.0–61.6
Missing	29.3	28.5–30.0
Types of STIs		
Gonorrhea	17.0	14.0–20.5
Trichomonads	6.7	4.8–9.2
Hepatitis	40.5	36.4–44.8
Chlamydia	28.7	17.3–47.1
HPV/Genital warts	5.9	4.2–8.3
Syphilis	4.8	3.3–7.0
Herpes	3.4	2.2–5.4
PID/Unspecified	18.3	15.6–22.3
# of Lifetime sex partners	2; IQR, 1–3	
Parity	3; IQR, 2–3	
History of smoking		
Yes	0.6	0.5–0.7
No	98.5	98.3–98.7
Missing	1.0	0.8–1.1
History of Alcohol		
Yes	6.5	6.1–6.9
No	92.5	92.1–93.0
missing	1.0	0.9–1.2
Race		
Black	99.7	99.6–99.8
Others	0.1	0.1–0.2
Missing	0.2	0.1–0.30
Cytology outcome at first CCS		
NILM	85.7	85.1–86.3
ASCUS	4.1	3.8–4.5
LSIL	5.6	5.3–6.0
ASCUS-H	1.6	1.4–1.8
AGUS	0.2	0.2–0.3
HSIL	2.5	2.3–2.8
HSIL, suspicion for invasion	0.2	0.2–0.3
Cytology category at first CCS		
Normal cervical cytology	85.7	85.1–86.3
Mild cervical dysplasia	9.7	9.3–10.2
Severe cervical dysplasia	4.6	4.2–4.9

*SD* standard deviation, *IQR* Interquartile range), % (Percent)

**Table 2:** Baseline socio-demographic characteristics by referral type in women at first CCS in an opportunistic screening program in Jos, Nigeria (*N* = 14,088)

Variable	Self-referral	Provider-referral	*p*-value
HIV status			0.001^b^
Not infected	6682 (50.8)	6473 (49.2)	
Infected	220 (31.3)	483 (68.7)	
Age at first CCS(Mean ± SD)	37.5 ± 10.1	38.6 ± 10.0	0.001^a^
No of Lifetime sex partners(Mean ± SD)	2.2 ± 1.8	2.2 ± 1.9	0.074^a^
Use of condom			
No	6304 (51.9)	5841 (48.1)	0.001^b^
Yes	398 (39.4)	611 (60.6)	
History of smoking			
No	6949 (50.1)	6926 (49.9)	0.001^b^
Yes	18 (22.8)	61 (77.2)	
History of Alcohol			
No	6542 (50.2)	6493 (49.8)	0.061^b^
Yes	428 (47.0)	483 (53.0)	
History of vaginal infection			
No	1154 (49.3)	1189 (50.7)	0.477^b^
Yes	5648 (50.1)	5625 (49.9)	
Ever diagnosed with STI			
No	4814 (56.2)	3747 (43.8)	0.001^b^
Yes	628 (44.8)	778 (55.3)	
Age at first sex	20.5 ± 3.9	19.8 ± 3.9	0.001^a^
Education years completed(Mean ± SD)	11.8 ± 2.9	11.7 ± 3.2	0.439^a^
Parity(Mean ± SD)	3.4 ± 2.4	3.7 ± 2.6	0.001^a^
Annual household income in USD(Mean ± SD)	4374.5 ± 4263.7	3971.7 ± 3851.2	0.001^a^

^a^Student t-test and ^b^Pearson’s chi^2^. Percent in parenthesis, SD (standard deviation)

Also, the unadjusted odds ratio for receiving a provider referral if HIV infected was 2.27 (95% CI: 1.92, 2.68) compared to being HIV uninfected. The unadjusted odds ratio for receiving a provider referral for other patient-reported socio-demographic and risk factors have been summarized in Table 3. In the final model, adjusting for smoking, age at first coitus < 22 years, age at first CCS ≥ 35 years, parity ≥5, history of condom use for sex, and 7–12 years of completed education, women who reported HIV infected were 2.35 times more likely to receive a provider referral for their first CCS compared to women who were HIV uninfected (aOR = 2.35; 95% CI: 1.95, 2.82, *p* = 0.001). Other socio-demographic factors that were independently associated with provider-referral for first cervical cancer screening were age ≥ 35 years (aOR = 1.25, 95% CI: 1.15, 1.35), parity ≥5 (aOR = 1.18, 95% CI: 1.09, 1.28), age at first sex ≤22 years (aOR = 1.27; 95% CI: 1.16, 1.39), smoking history (aOR = 3.20; 95% CI: 1.67, 6.12) and use of condoms (aOR = 1.47; 95% CI: 1.28, 1.70). We also found that women who reported completing 7–12 years (grade 7 to high-school) of education were less likely to receive a provider-referral than women with less than 7 years (equivalent to grade 6 or less) of completed education (aOR = 0.77; 95% CI: 0.71, 0.84). These results have been summarized in Table 3.

**Table 3:** Bivariable and multivariable logistic regression with unadjusted and adjusted odds ratio of the association between patient-reported HIV status, other socio-demographic factors and provider-referral for CCS at first screening in Jos, Nigeria (*N* = 14,088)

Variable	OR (95% CI)	p-value	aOR (95% CI)	P-value
HIV status				
Uninfected	1.0			
Infected	2.27 (1.93, 2.67)	0.001	2.35 (1.95, 2.82)	0.001
Age in years				
< 35 years	1.0			
≥ 35 years	1.34 (1.25, 1.43)	0.001	1.25 (1.15, 1.35)	0.001
Education (years completed)				
< 7 years	1.0			
7-12 years	0.65 (0.57, 0.73)	0.001	0.77 (0.71, 0.84)	0.001
> 12 years	0.81 (0.72, 0.90)	0.001	–	–
Parity				
< 5	1.0			
≥ 5	1.27 (1.18, 1.36)	0.001	1.18 (1.09, 1.28)	0.001
Age at first sex				
> 22 years	1.0			
≤ 22 years	1.38 (1.28, 1.49)	0.001	1.27 (1.16, 1.39)	0.001
Total life-time sex partners				
< 3	1.0			
≥ 3	1.05 (0.97, 1.14)	0.234	–	–
Use of condoms during sex				
No	1.0			
Yes	1.66 (1.45, 1.89)	0.001	1.47 (1.28, 1.70)	0.001
History of vaginal infection				
No	1.0			
Yes	0.97 (0.89, 1.06)	0.477	–	–
Ever diagnosed with STIs				
No	1.0			
Yes	1.59 (1.42, 1.78)	0.001	–	–
History of Smoking				
No	1.0			
Yes	3.40 (2.01, 5.76)	0.001	3.20 (1.67, 6.12)	0.001
Alcohol consumption				
No	1.0			
Yes	1.14 (0.99, 1.30)	0.061	–	–

Hosmer-Lemeshow goodness-of-fit p-value = 0.223, LR (chi2) = 275.9, Pseudo R^2^ = 0.0186

## Discussion

The principal findings in this analysis showed that women who reported being HIV infected were more than 2 times more likely to be referred by a provider at the time of first CCS compared to women who were HIV uninfected (aOR = 2.35; 95% CI: 1.95, 2.82). We also found that women who had completed at least 7–12 years of education were less likely to received provider-referral compared to women of lower educational attainment (aOR = 0.77; 95% CI: 0.71, 0.84). Other socio-demographic factors that were significantly associated with provider-referral for first CCS were age ≥ 35 years, parity ≥5, age at first sex ≤22 years, smoking history, and use of condoms.

The finding that patient-reported HIV was significantly associated with provider-referral at first CCS is particularly important since several studies have found that HIV infected women are at greater risk of developing precancer and ICC [21, 30–34]. Because of this risk, studies have demonstrated the critical role of health care providers in linking HIV infected women to important reproductive health services including CCS in areas with a high HIV burden [35]. The finding that patient-reported HIV was associated with provider-referral in our study population is not surprising giving that the OSCC is located in one of the tertiary health institutions supported by Presidential Emergency Plan for AIDS Relief (PEPFAR) and one of the largest facilities that offers HIV care and treatment in West Africa. It is possible that providers offering HIV care to these women are aware of both the risk of cervical cancer in these women and the availability of screening services in the facility, thereby offering referral to such high-risk population. Prior reports on the role of medical care providers facilitating CCS for HIV infected women receiving care in the same facility with a gynecologic care provider has been well documented in a U.S. HIV population [36], and it has been recommended that HIV care be integrated with gynecologic care, and educating clinicians to recommend CCS to these women could significantly improve adherence and utilization of CCS [37, 38].

Although, the prevalence of smoking is reportedly very low (0.6%) in our study population, women with reported history of smoking were more likely to have received a provider-referral for their first CCS. This could be related to provider’s knowledge that smoking is a risk factor for cervical cancer [39–43] thereby initiating counselling and offering screening referrals for such high-risk women. Other demographic factors such as age and parity have been documented in previous studies as epidemiologic risk factors for cervical precancer and progression to ICC [44]. These demographic factors were also found to be significantly associated with a higher likelihood for receiving a provider-referral at the time of first CCS in our study population.

The finding that more educated women were less likely to receive provider-referral for first CCS is interesting. This possibly implies that more educated women were more likely to be aware of CCS and more likely to self-refer themselves for screening compared to the less educated. This explanation has some plausibility given the findings of recent systematic review of qualitative studies on the barriers to utilization of Pap screening in sub-Saharan Africa [45]. The systematic review reported client factors such as lack of knowledge and awareness about Pap smear among the barriers for low utilization of Pap screening [45]. Also related to provider-referral, the study found that provider barriers such as failure to inform or encourage women to screen were important provider factors contributing to low Pap utilization in sub-Saharan Africa [45]. A related systematic review also recommended improvement in cervical health education, addressing cultural beliefs and practices, spousal support, provider attitude and addressing the problems of cost and physical access to CCS services as interventions to improve screening utilization in sub-Saharan Africa [46].

Our findings suggest that women with known potential risk factors for cervical cancer such as HIV infection, multiparity, and smoking were more likely to be referred for screening by providers. This is an important finding for opportunistic CCS, and implementation of screening guidelines in such settings should encourage providers to assess potential risk factors in women accessing routine clinical care and those with such reported risk factors should be given screening referrals and encouraged to receive CCS. Studies have also identified that interventions that increased discussions between providers and women, educating women on the benefits of screening, and allaying their fears on possible screening outcomes are significantly associated with participation in screening [47].

The strength of our study findings is related to the relatively large sample size of our study population spanning a decade of CCS services offered in an opportunistic setting in a cosmopolitan Nigerian city that also offers care to a large population of HIV infected adults in West Africa. To the best of our knowledge this is the first secondary analysis of CCS data in Nigeria focusing on understanding the contributions of providers in utilizing CCS services. We feel that our findings could be generalized to other settings in West Africa with ongoing HIV care and availability of opportunistic CCS services. We however, recognize and acknowledge the limitations of self-reported risk factors in this analysis. It is possible that some women may have concealed some information that could affect the internal validity of our estimates.

Our future research will focus on elucidating provider and patient perspectives on the facilitators and barriers to CCS in an opportunistic screening setting using qualitative research methodology. For instance, it will be appropriate to understand the perspectives of providers on implementation of CCS guidelines in Nigeria and the factors that could enhance adherence to such guidelines in various practice settings. It is also important to understand patient perspectives on the acceptability of male providers performing pelvic examinations and alternative screening methods such as self-sampling for HPV testing in various cultural settings in Nigeria.

## Conclusions

Women who reported having known risk factors for cervical cancer such as HIV infection, multiparity, and smoking are significantly more likely to received recommendation and referral for CCS from a health care provider during routine clinical care visits. More educated women with at least 7–12 years of education are more likely to be aware of CCS service and self-refer for screening than women who have lower education. Despite the limitations identified in this secondary data analysis, healthcare providers in Nigeria should be encouraged and supported to make CCS counseling, recommendations and referral during routine clinical care to eligible women who have not had a CCS. This is particularly important in the current Nigerian setting where HPV vaccination is not supported and the opportunity for screening is largely dependent on provider-initiated counseling and screening either by the provider or through referral to facilities offering a screening service.

## Additional files

Additional file 1: The operational definition of independent variables and the primary outcome variables (DOCX 16 kb)

Additional file 2: STROBE Statement—Checklist of items that should be included in reports of *cross-sectional studies*. (DOC 86 kb)

## Abbreviations

AIDS: Acquired Immune Deficiency Syndrome
aOR: Adjusted Odds Ratio
AORTIC: African Organization for Research and Training in Cancer
CCS: Cervical Cancer Screening
CI: Confidence Interval
HBM: Health Belief Model
HIV: Human Immunodeficiency Virus
HPV: Human Papillomavirus
ICC: Invasive Cervical Cancer
IQR: Interquartile Range
LMICs: Low and Middle-Income Countries
NIH: National Institutes of Health
OR: Odds Ratio
OSCC: Operation Stop Cervical Cancer
SD: Standard Deviation
STIs: Sexually Transmitted Infections
WHO: World Health Organization
