# Supplementary material for: Association between patient-reported HIV status and provider recommendation for screening in an opportunistic cervical Cancer screening setting in Jos, Nigeria
Source: BMC Health Serv Res. 2018 Nov 22;18:885. doi: 10.1186/s12913-018-3700-y (PMC6251217; doi:10.1186/s12913-018-3700-y)
Supplement: Supplementary file 2 — STROBE Statement—Checklist of items that should be included in reports of cross-sectional studies. (DOC 86 kb) [file 12913_2018_3700_MOESM2_ESM.doc]

**Additional File 2:**

STROBE Statement—Checklist of items that should be included in reports of ***cross-sectional studies***

|  | Item No | Recommendation |  |
| --- | --- | --- | --- |
| **Title and abstract**  ***Reported on pages 1 and 2*** | 1 | (*a*) Indicate the study’s design with a commonly used term in the title or the abstract |  |
|  |  | (*b*) Provide in the abstract an informative and balanced summary of what was done and what was found |  |
| Introduction | | |  |
| Background/rationale  ***Reported on pages 3-5*** | 2 | Explain the scientific background and rationale for the investigation being reported |  |
| Objectives  ***Reported in first paragraph on page 5*** | 3 | State specific objectives, including any prespecified hypotheses |  |
| Methods | | |  |
| Study design  ***Reported on page 6, first paragraph under study design and setting*** | 4 | Present key elements of study design early in the paper |  |
| Setting  ***Reported on page 6, first paragraph under study design and setting*** | 5 | Describe the setting, locations, and relevant dates, including periods of recruitment, exposure, follow-up, and data collection |  |
| Participants  ***Reported on page 7, under study sample.*** | 6 | (*a*) Give the eligibility criteria, and the sources and methods of selection of participants |  |
| Variables  ***Reported on page 7, under key independent variables and outcomes*** | 7 | Clearly define all outcomes, exposures, predictors, potential confounders, and effect modifiers. Give diagnostic criteria, if applicable |  |
| Data sources/ measurement  ***Reported on pages 6 and 7 under methods section*** | 8* | For each variable of interest, give sources of data and details of methods of assessment (measurement). Describe comparability of assessment methods if there is more than one group |  |
| Bias  ***Reported on pages 7 and 8 under statistical analyses section*** | 9 | Describe any efforts to address potential sources of bias |  |
| Study size  ***Reported on page 7 and appendix 1 file attached*** | 10 | Explain how the study size was arrived at |  |
| ***Quantitative*** ***variables***  ***Reported on pages 7 and 8 under statistical analyses section*** | 11 | Explain how quantitative variables were handled in the analyses. If applicable, describe which groupings were chosen and why |  |
| Statistical methods  ***Reported on pages 7 and 8 under statistical analyses section.***  ***Sensitivity analyses were not applicable and not done in this study*** | 12 | (*a*) Describe all statistical methods, including those used to control for confounding |  |
|  |  | (*b*) Describe any methods used to examine subgroups and interactions |  |
|  |  | (*c*) Explain how missing data were addressed |  |
|  |  | (*d*) If applicable, describe analytical methods taking account of sampling strategy |  |
|  |  | (*e*) Describe any sensitivity analyses |  |
| Results | | |  |
| Participants  ***Reported first paragraph of results section on page 9 and in appendix 1*** | 13* | (a) Report numbers of individuals at each stage of study—eg numbers potentially eligible, examined for eligibility, confirmed eligible, included in the study, completing follow-up, and analysed |  |
|  |  | (b) Give reasons for non-participation at each stage |  |
|  |  | (c) Consider use of a flow diagram |  |
| Descriptive data  ***Reported under results section and supplementary tables 1 and 2, and supplementary plots attached*** | 14* | (a) Give characteristics of study participants (eg demographic, clinical, social) and information on exposures and potential confounders |  |
|  |  | (b) Indicate number of participants with missing data for each variable of interest |  |
| Outcome data  ***Reported in result section on pages 9 to 10*** | 15* | Report numbers of outcome events or summary measures |  |
| Main results  ***Reported in result section on pages 9 to 10*** | 16 | (*a*) Give unadjusted estimates and, if applicable, confounder-adjusted estimates and their precision (eg, 95% confidence interval). Make clear which confounders were adjusted for and why they were included |  |
|  |  | (*b*) Report category boundaries when continuous variables were categorized |  |
|  |  | (*c*) If relevant, consider translating estimates of relative risk into absolute risk for a meaningful time period |  |
| Other analyses  N/A | 17 | Report other analyses done—eg analyses of subgroups and interactions, and sensitivity analyses |  |
| Discussion | | |  |
| Key results  **Reported in first paragraph of discussion on page 10** | 18 | Summarise key results with reference to study objectives |  |
| Limitations  ***First paragraph on page 13 under the discussion section*** | 19 | Discuss limitations of the study, taking into account sources of potential bias or imprecision. Discuss both direction and magnitude of any potential bias |  |
| Interpretation  ***Reported on pages 11 to 13*** | 20 | Give a cautious overall interpretation of results considering objectives, limitations, multiplicity of analyses, results from similar studies, and other relevant evidence |  |
| Generalisability  ***Reported in the third sentence, first paragraph on page 13*** | 21 | Discuss the generalisability (external validity) of the study results |  |
| Other information | | |  |
| Funding  ***Reported on page 16 in the Funding section*** | 22 | Give the source of funding and the role of the funders for the present study and, if applicable, for the original study on which the present article is based |  |

*Give information separately for exposed and unexposed groups.

**Note:** An Explanation and Elaboration article discusses each checklist item and gives methodological background and published examples of transparent reporting. The STROBE checklist is best used in conjunction with this article (freely available on the Web sites of PLoS Medicine at http://www.plosmedicine.org/, Annals of Internal Medicine at http://www.annals.org/, and Epidemiology at http://www.epidem.com/). Information on the STROBE Initiative is available at www.strobe-statement.org.
